# First Transcriptome and Digital Gene Expression Analysis in Neuroptera with an Emphasis on Chemoreception Genes in *Chrysopa pallens* (Rambur)

**DOI:** 10.1371/journal.pone.0067151

**Published:** 2013-06-27

**Authors:** Zhao-Qun Li, Shuai Zhang, Yan Ma, Jun-Yu Luo, Chun-Yi Wang, Li-Min Lv, Shuang-Lin Dong, Jin-Jie Cui

**Affiliations:** 1 State Key Laboratory of Cotton Biology, Institute of Cotton Research of CAAS, Anyang, China; 2 Education Ministry Key Laboratory of Integrated Management of Crop Diseases and Pests, College of Plant Protection, Nanjing Agricultural University, Nanjing, China; University of California Davis, United States of America

## Abstract

**Background:**

*Chrysopa pallens* (Rambur) are the most important natural enemies and predators of various agricultural pests. Understanding the sophisticated olfactory system in insect antennae is crucial for studying the physiological bases of olfaction and also could lead to effective applications of *C. pallens* in integrated pest management. However no transcriptome information is available for Neuroptera, and sequence data for *C. pallens* are scarce, so obtaining more sequence data is a priority for researchers on this species.

**Results:**

To facilitate identifying sets of genes involved in olfaction, a normalized transcriptome of *C. pallens* was sequenced. A total of 104,603 contigs were obtained and assembled into 10,662 clusters and 39,734 singletons; 20,524 were annotated based on BLASTX analyses. A large number of candidate chemosensory genes were identified, including 14 *odorant-binding proteins* (*OBPs*), 22 *chemosensory proteins* (*CSPs*), 16 *ionotropic receptors,* 14 *odorant receptors*, and genes potentially involved in olfactory modulation. To better understand the OBPs, CSPs and cytochrome P450s, phylogenetic trees were constructed. In addition, 10 digital gene expression libraries of different tissues were constructed and gene expression profiles were compared among different tissues in males and females.

**Conclusions:**

Our results provide a basis for exploring the mechanisms of chemoreception in *C. pallens*, as well as other insects. The evolutionary analyses in our study provide new insights into the differentiation and evolution of insect OBPs and CSPs. Our study provided large-scale sequence information for further studies in *C. pallens*.

## Background

A sophisticated olfactory system is a key physiological trait for insect survival and reproduction. Olfaction is crucial to many behaviors, including feeding, mating, toxin avoidance, and negative taxis [1]. Previous studies have shown that the major olfactory-related proteins in insects are odorant-binding proteins (OBPs), odorant receptors (ORs), ionotropic receptors (IRs), sensory neuron membrane proteins (SNMPs) and odorant-degrading enzyme (ODEs) [2]. The process of insect olfaction can be generalized as having several main steps. First, odorants reaching the pore tubules enter a sensillum and are bound and solubilized by OBPs; then, the OBPs are transported through the sensillar lymph that fills the cavity around the dendrites, where they finally activate membrane-bound ORs [2]. After OR activation, the odorant will be rapidly removed by ODEs to restore the sensitivity of the sensory neuron [3], [4].

In addition, some other proteins are also thought to be involved in insect olfaction. Soluble chemosensory proteins (CSPs) are also found in abundance within the sensillar lymph. Similar to OBPs, CSPs are thought to affect insect chemoreception by enhancing the solubility of semiochemicals and delivering them to the chemosensory receptors [5]. SNMPs, located in the dendritic membranes of pheromone sensitive neurons [4], [6], appear to trigger ligand delivery to the olfactory receptor [7]. To maintain olfactory system sensitivity, ODEs then remove the odorant. Carboxylesterases (CXEs) and aldehyde oxidases (AOXs), which degrade ester and aldehyde pheromones [8], [9], were the first identified ODEs in insects [10], [11]. Additionally, P450s play important roles in many physiological functions in insects, such as signal molecule metabolism, adaptation to host plants, and insecticide resistance. However, some reports have revealed that P450s may be involved in odorant processing in antennae [12]–[14].

Most Neuroptera are natural enemies of agroecosystem pests. The lacewing *Chrysopa pallens* (Rambur) (Neuroptera: Chrysopidae) are the most important natural predators of various pests such as aphids, coccids, thrips, mites, and caterpillars [15]. The larvae of *C. pallens* are called aphid-lions because each can eat more than 800 aphids during its larval stage. *C. pallens* are voracious predators that adapt well to different agroecosystems [16]–[18], so they are very useful auxiliaries in integrated pest management (IPM). Mass production and release of *C. pallens* in fields is economically unfeasible compared with recruitment of existing individuals in the environment. Based on the feeding habits of adult lacewings, several plant-derived compounds were suggested as attractants [19]–[22]. Recently, individual aphid sex pheromone compounds (enantiomers of nepetalactol and nepetalactone) were shown to attract predatory males of *Chrysopa* spp. and can potentially be used to enhance biological control of aphids [23]. Furthermore, field attraction of *Chrysopa* lacewings to (1*R*, 4a*S*, 7*S*, 7a*R*)-nepetalactol has been reported [24], [25].

To enhance the effectiveness of *C. pallens* in IPM via tools such as the “push-pull” strategy, in which combinations of repellent and attractive stimuli are used to alter the populations of insect pests and natural enemies [26], research about olfactory behavior, including hunting, mating, and oviposition, is essential. Discovery of chemoreception genes is the primary step for exploring the mechanism underlying insect olfactory behavior. Unfortunately, sequence data from *C. pallens* is scarce. Therefore, more sequence information and transcriptome analyses could serve as valuable molecular resources leading ultimately to more effective use of *C. pallens* in pest control and contributing to the understanding of co-evolution between *C. pallens* and its prey.

Deep sequencing data can provide extensive information about genomes and gene expression profiling via next-generation high-throughput techniques. For example, Illumina technology have proven to be an efficient and low cost method to find genes in many insects, such as *Spodoptera littoralis*, *Manduca sexta*, *Helicoverpa armigera*, *Apis cerana cerana*, *Cnaphalocrosis medinalis*, and *Nilaparvata lugens* [27]–[33]. High-throughput sequencing has been successfully used to identify olfactory genes in Lepidoptera [30], [34]–[36], but no transcriptome information has been reported from Neuroptera.

Given the lack of sequence information for *C. pallens*, we constructed a whole-body cDNA library and obtained 50,396 distinct unigenes by Illumina RNA sequencing and sequence assembly. In total, 14 *OBPs*, *22 CSPs*, 60 *P450s*, 16 *IRs*, 14 *ORs*, 2 *SNMPs*, 54 *CXEs* and 21 *AOXs* were obtained. Furthermore, 10 digital gene expression (DGE) libraries were constructed and the gene expression profiles were compared among different tissues.

## Results

### Illumina Sequencing and Sequence Assembly

**Figure 1:**
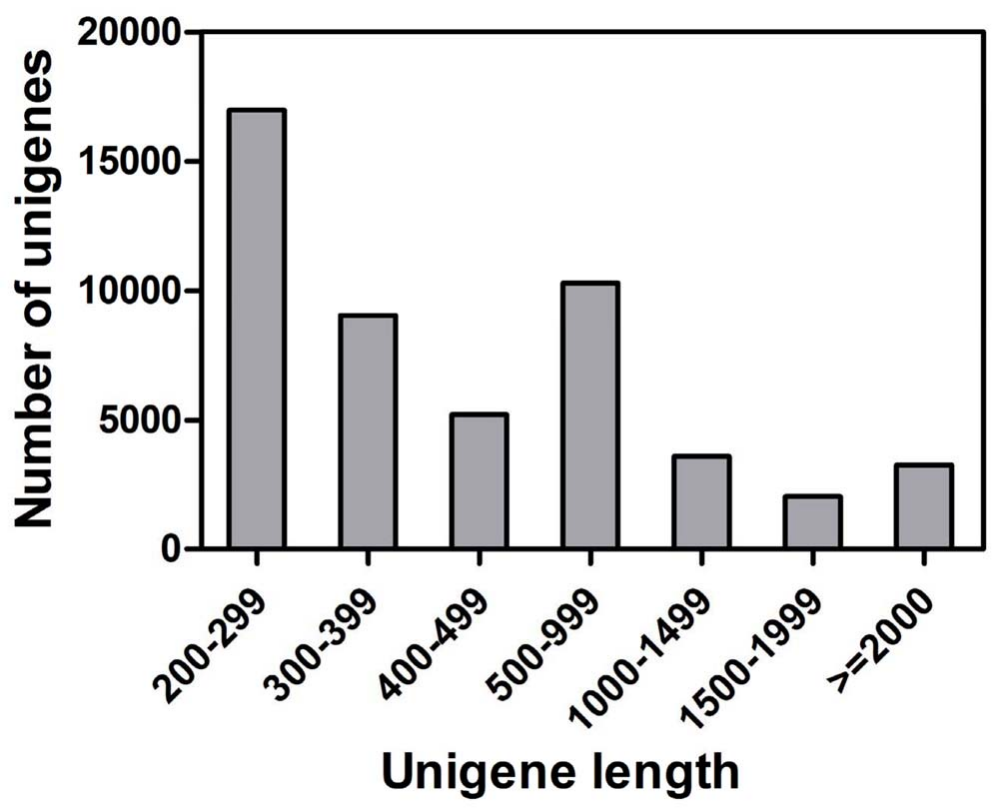
Length distribution of *Chrysopa pallens* unigenes. All unigene sizes were calculated. The X-axis shows the length (nt) of unigenes. The Y-axis shows the number of unigenes.

A library of *C. pallens* was constructed by Illumina sequencing. A total of 58,580,430 raw reads were obtained from the sample. After removing low-quality, adaptor, and contaminating sequences, 54,331,274 clean reads (Q_20_ = 98.87%) with an accumulated length of 4,889,814,660 nucleotides (nt) were generated and assembled into 104,603 contigs with an N_50_ length of 567 nt. Using paired-ends reads, these contigs were further assembled into 50,396 distinct unigenes, including 10,662 clusters and 39,734 singletons, with a mean length of 722 nt and N_50_ of 1142 bp (Table 1). The size distribution indicated that the 10,107 unigenes were longer than 1000 bp (Fig. 1).

**Table 1:** Summary of *Chrysopa pallens* transcriptome.

	Number
**Total number of raw reads**	58,580,430
**Total number of clean reads**	54,331,274
**Total nucleotides**	4,889,814,660
**Q_20_ percentage**	98.87%
**GC percentage**	37.95%
**Total number of contigs**	104,603
**Mean length of contigs**	340
**N_50_ of contigs (nt)**	567
**Total number of unigenes**	50,396
**Mean length of unigenes**	722
**N_50_ of unigenes (nt)**	1,142
**Number of distinct clusters**	10,662
**Number of distinct singletons**	39,734

### Gene Identification and Functional Annotation

For annotation, all of 50,396 distinct unigenes longer than 200 bp were analysised by searching against NR, Swiss-Prot, KEGG, COG, and GO databases using BLASTX with a cut-off e-value of 10^−5^. A total of 21,644 unigenes (42.96%) returned a BLAST result; 20,524, 16,967, 14,938, 8,154, and 6,209 were annotated by NR, Swiss-Prot, KEGG, COG, and GO, respectively. A much higher cut-off e-value of 10^−20^ were also used to search against NR, Swiss-Prot, KEGG and COG databases by BLASTX, and 15584, 12051, 10109 and 3774 unigenes were annotated, respectively.

**Figure 2:**
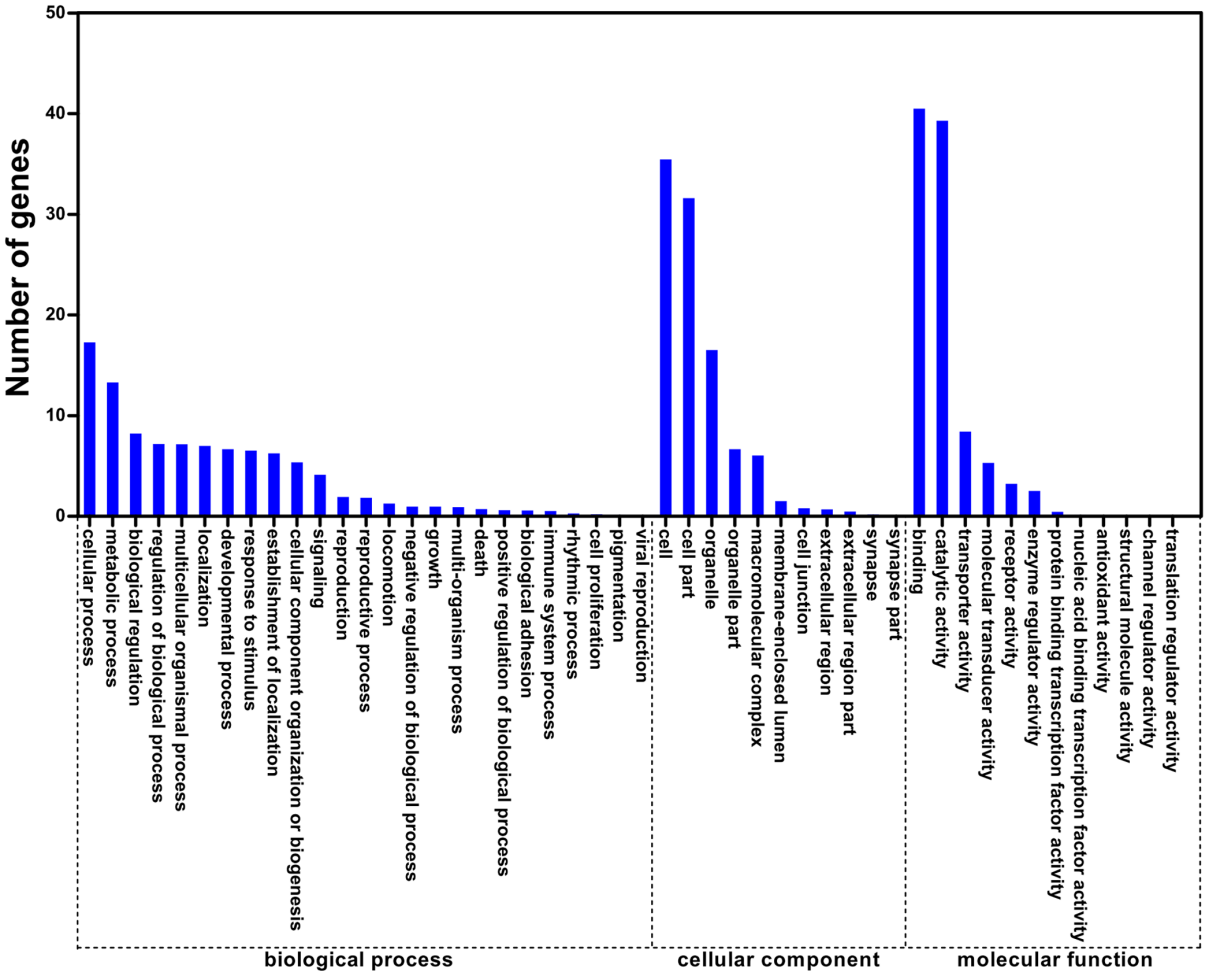
Distribution of *Chrysopa pallens* unigenes annotated by GO. The X-axis shows three categories and their subcategories. The Y-axis shows the percentage of sequences. The analysis level was 3.

GO annotation was used to classify the function of the *C. pallens* unigenes. Among the distinct unigenes, 6,209 (12.33%) corresponded to at least one GO term. A total of 14,790, 8,609, and 5,706 unigenes were classified as being involved in the categories of biological process, cellular component, and molecular function, respectively. Within biological process, cellular process and metabolic process represented the most abundant GO terms. Most unigenes that corresponded to cellular component were involved in cell and cell part. Binding and catalytic activity were most prevalent in of molecular function (Fig. 2).

**Figure 3:**
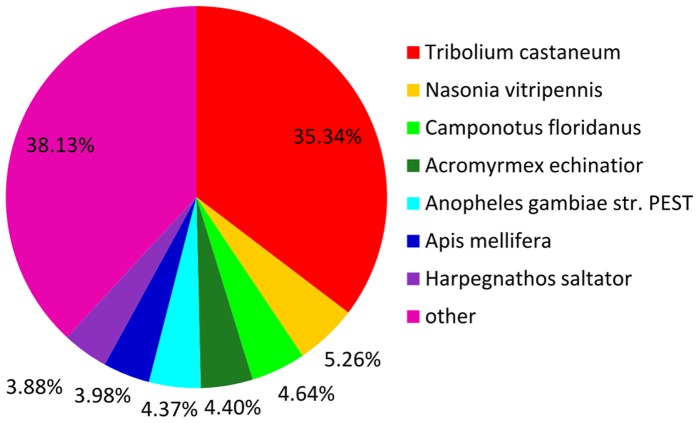
Species distribution of the BLASTX results. Unigenes were searched against the NR protein database using BLASTX with a cutoff e-value <10^−5^ and the proportions of each species (represented by different colors) graphed. Species with proportions of more than 1% are shown.

To further evaluate the functions of the unigenes, COG annotation was used. A total of 8,154 of the unigenes were distributed into 25 COG classifications. Among these, the cluster “general function prediction only” was the biggest group. The clusters “replication, recombination and repair”, “transcription” and “translation, ribosomal structure and biogenesis” were also enriched (Fig. S1). The species distribution of unigenes was annotated with the NR protein database. The *C. pallens* sequences revealed substantial (35.34%) matches with *Tribolium castaneum* (Fig. 3).

We identified 14 candidate *OBPs*, 22 candidate *CSPs*, 60 candidate *P450s*, 16 candidate *IRs*, 14 candidate *ORs*, 2 candidate *SNMPs*, 54 candidate *CXEs*, and 21 candidate *AOXs* with BLASTX and BLASTN (Table S1).

Additionally, 36 putative chemoreception genes, including 14 *OBPs* and 22 *CSPs*, were used to confirm transcriptome assemblies by sequencing their PCR products. The sequences obtained from positive clones had ≥99% identities at the nucleic acid level with corresponding sequences from the transcriptome, indicating that unigene assemblies were adequate.

### Sequence Alignment and Phylogenetic Analyses

#### *OBP* genes

**Figure 4:**
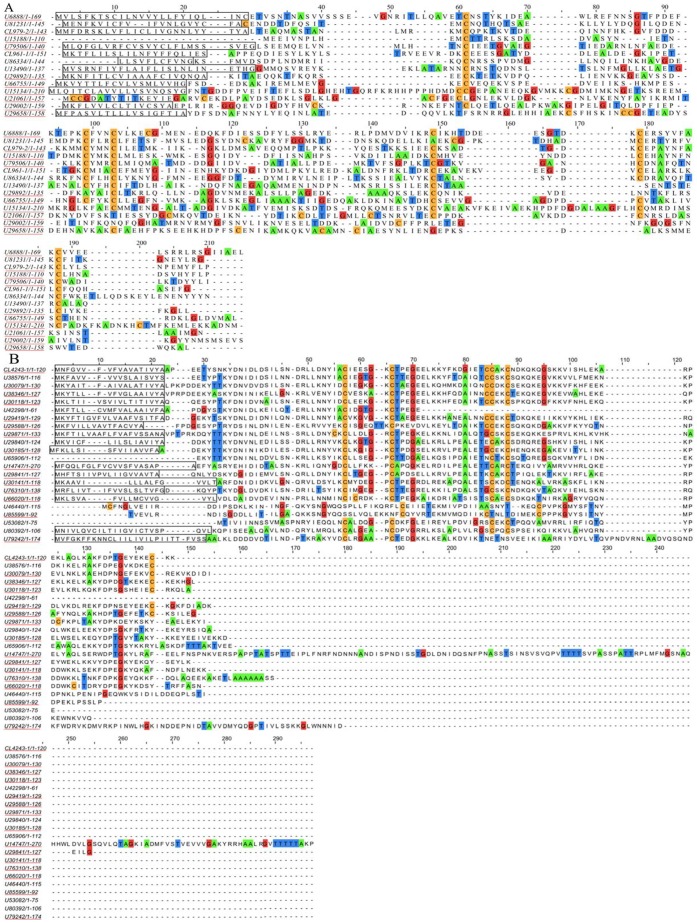
Alignment of amino acid sequences of the candidate OBPs and CSPs. (A): Alignment of amino acid sequences of the candidate OBPs. (B): Alignment of amino acid sequences of the candidate CSPs. Predicted signal peptides are boxed, and the conserved cysteines are highlighted in blue. Sequences which contained a full length open reading frame were labeled by red line.

**Figure 5:**
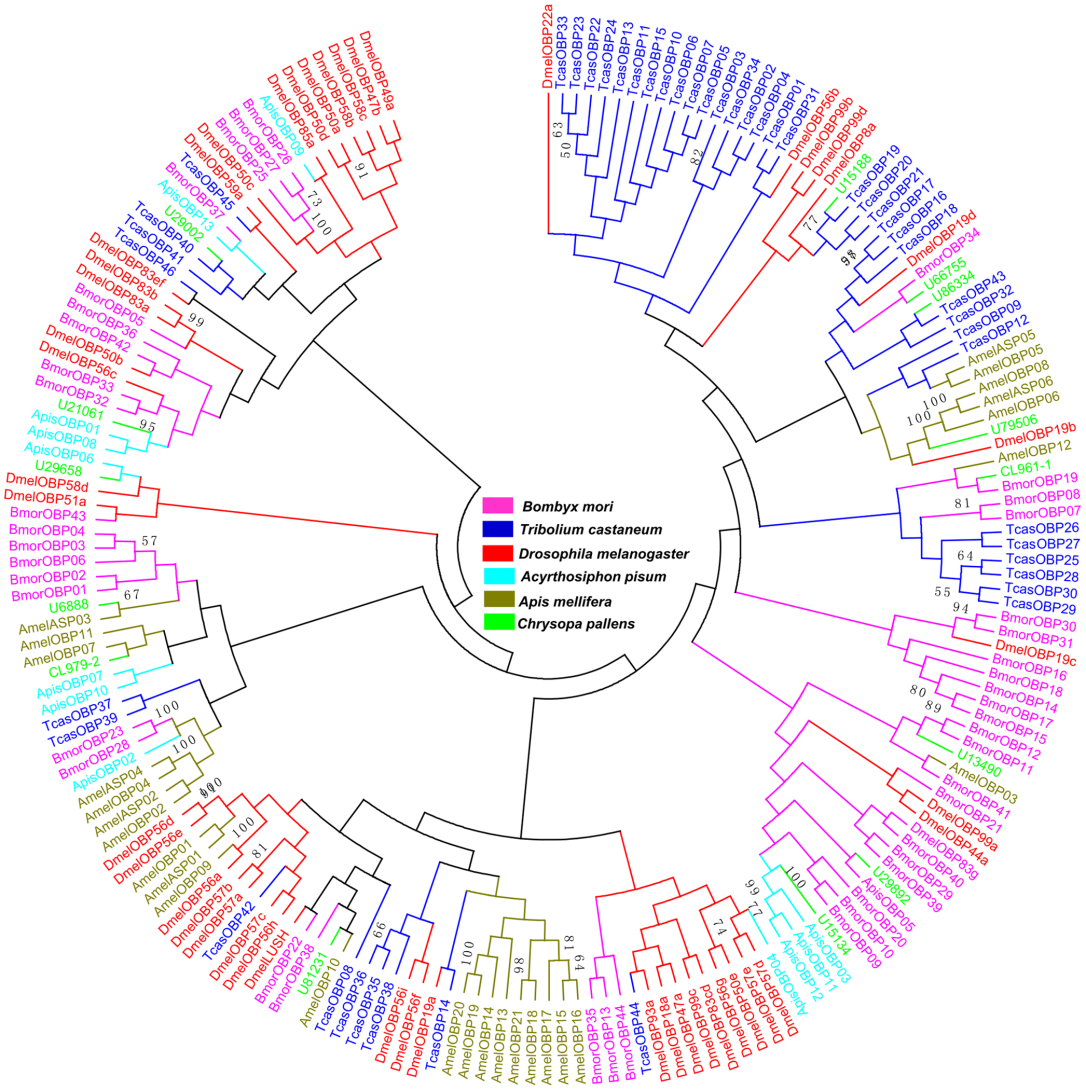
Phylogenetic tree of candidate odorant-binding proteins (OBPs) from ***Chrysopa pallens*** **and five other species.** Tcas: *Tribolium castaneum*, Dmel: *Drosophila melanogaster*, Bmor: *Bombyx mori*, Apis: *Acyrthosiphon pisum*, Amel: *Apis mellifera*. The *C. pallens* unigenes were labeled with ‘CL’ (clusters) and ‘U’ (unigenes). CL-X: CL-contigX.

All unigenes were searched by BLASTX, 14 distinct unigenes encoding OBPs were identified from the *C. pallens* transcriptome. Based on sequence analysis, 10 sequences contained a full length open reading frame with a predicted signal peptide sequence (Fig. 4A). These 14 genes were used to construct a phylogenetic tree with other OBPs from five species (Fig. 5). The tree revealed that all the candidate OBP sequences were distributed among orthologous groups. Three of them showed higher homology to ApisOBPs.

#### *CSP* genes

**Figure 6:**
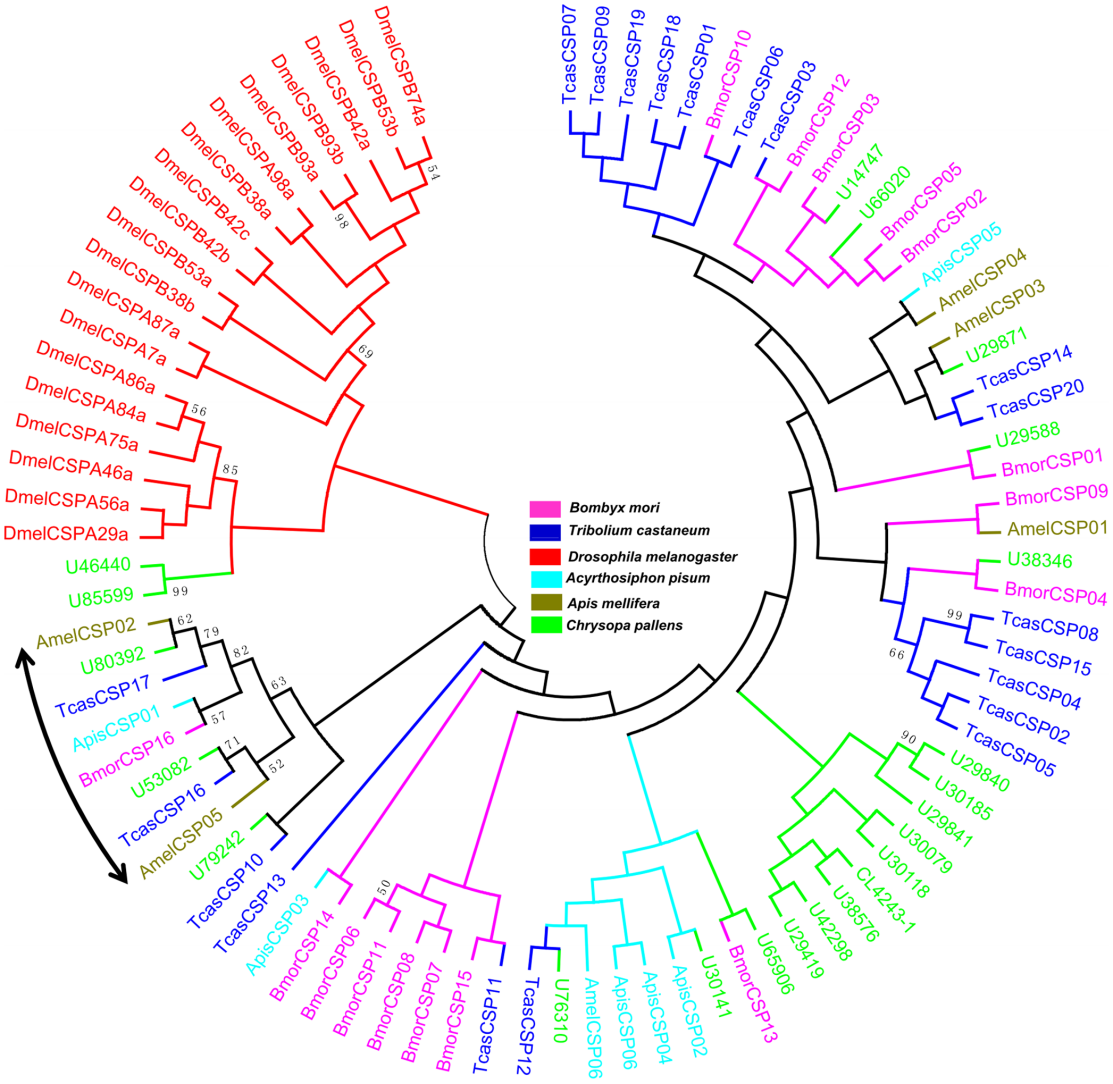
Phylogenetic tree of candidate chemosensory proteins (CSPs) from *Chrysopa pallens* and five other species. Tcas: *Tribolium castaneum*, Dmel: *Drosophila melanogaster*, Bmor: *Bombyx mori*, Apis: *Acyrthosiphon pisum*, Amel: *Apis mellifera*. The *C. pallens* unigenes were labeled with ‘CL’ (clusters) and ‘U’ (unigenes). CL-X: CL-contigX. Arrow means 8 CSPs representing five of the six species clustered together with a bootstrap percentage >50.

Sequence annotation led to the identification of 22 different candidate CSPs. Fifteen were predicted to have full lengths with signal peptides by sequence analysis (Fig. 4B). These 22 candidate CSPs were phylogenetically analyzed with data from five other species. Most CSPs from the same species formed monophyletic groups, unlike the OBPs. Nevertheless, eight CSPs representing five of the six species clustered together with a bootstrap percentage >50 (Fig. 6).

#### *P450* genes

**Figure 7:**
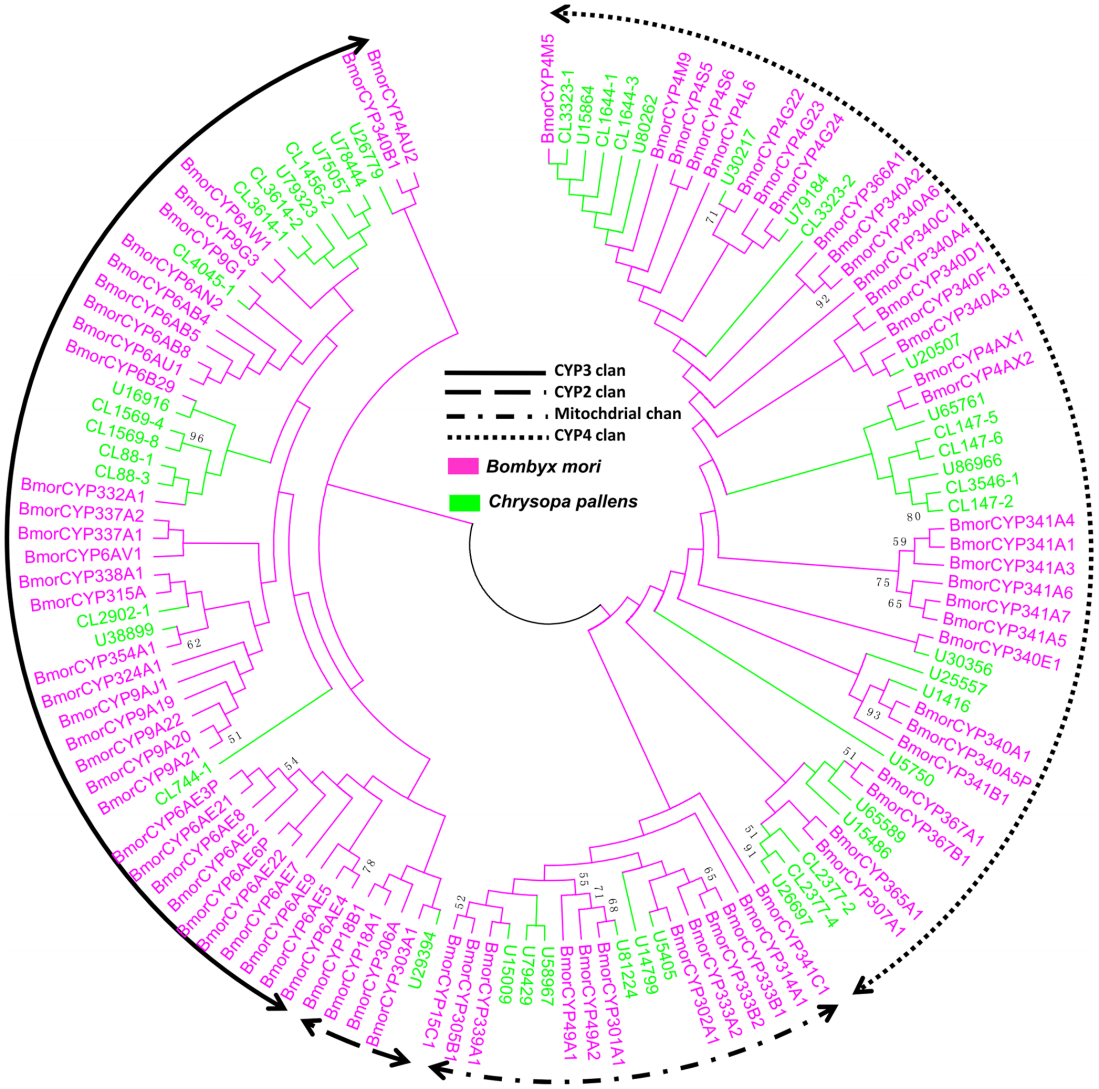
Phylogenetic tree of candidate cytochrome P450s from *Chrysopa pallens* and *Bombyx mori*. The *C. pallens* unigenes were labeled with ‘CL’ (clusters) and ‘U’ (unigenes). Bmor: *B. mori*. CL-X: CL-contigX.

The 134 putative P450 sequences in the *C. pallens* transcriptome assembly were identified based on similarity to known insect P450s. Forty-seven P450s (containing 28 full-length sequences) more than 220 amino acids in length were used to construct a phylogenetic tree with 82 *B. mori* P450s (Fig. 7). The 47 predicted P450 belonged to four major clades of P450s [37], namely the CYP2, CYP3, CYP4, and mitochondrial clades. Most belonged to CYP3 (22 candidates) and CYP4 (26).

### DGE Library Sequencing

Ten DGE libraries of *C. pallens* including, female antennae (FA), female heads without antennae (FH), female legs (FL), female wings (FW), female thoraxes and abdomens (FB), male antennae (MA), male heads without antennae (MH), male legs (ML), male wings (MW), and male thoraxes and abdomen (MB), were constructed to investigate the expression profiles of the unigenes in these tissues. In total, 5.76, 5.65, 6.25, 5.82, 5.80, 5.61, 5.28, 5.27, 5.80, and 5.63 million raw tags were generated in each library. The percentage of clean tags among the raw tags was more than 95% in each library (Fig. S2), and the percentages of clean tags that could be mapped to unigenes ranged from 69.05–79.15% (Table 2).

**Table 2:** Tag analysis statistics of *Chrysopa pallens* transcriptome.

Summary		Tissue				
		antenna	body^3^	head	leg	wing
Total raw data	Female	5759655	5797561	5652300	6247713	5815595
	Male	5614292	5625370	5278529	5265384	5797437
Distinct raw data	Female	201804	260245	213145	184043	199779
	Male	189924	179199	184821	169576	195394
Total clean tags	Female	5578201	5552846	5379205	6065829	5604779
	Male	5422631	5376574	5096997	5076253	5605875
Distinct clean tag	Female	83838	108157	87545	77603	82132
	Male	79548	72274	78626	68830	80616
All tags mapping to genes	Female	3852874	3934109	4009853	4720243	3869649
	Male	3854895	4123339	3797634	4017871	3920390
All tags mapping to genes^1^	Female	69.07	70.85	74.54	77.82	69.04
	Male	71.09	76.69	74.51	79.15	69.93
Distinct tags mapping to Genes	Female	38853	51146	43389	39611	34238
	Male	36804	36143	39242	35134	38068
Distinct tags mapping to Genes^1^	Female	46.34	47.29	49.56	51.04	41.69
	Male	46.27	50.01	49.91	51.04	47.22
Unambiguous tags mapping to genes	Female	3475421	3414969	3429608	3900638	3433164
	Male	3443210	3583915	3280687	3375695	3409000
Unambiguous tags mapping to genes^1^	Female	62.30	61.50	63.76	64.31	61.25
	Male	63.50	66.66	64.37	66.50	60.81
Distinct unambiguous tags mapping to genes	Female	33152	43132	36658	33421	29460
	Male	31539	30621	33143	29734	32542
Distinct unambiguous tags mapping to genes^1^	Female	39.54	39.88	41.87	43.07	35.87
	Male	39.65	42.37	42.15	43.20	40.37
All tag-mapped genes	Female	18369	22715	20058	18196	16619
	Male	18056	17612	18836	17016	17888
All tag-mapped genes^2^	Female	36.45	45.07	39.80	36.11	32.98
	Male	35.83	34.95	37.38	33.76	35.49
Unambiguous tag-mapped genes	Female	13833	17581	15173	13650	12558
	Male	13636	13228	14142	12747	13552
Unambiguous tag-mapped genes^2^	Female	27.45	34.89	30.11	27.09	24.92
	Male	27.06	26.25	28.06	25.29	26.89
Unknown tags	Female	1725327	1618737	1369352	1345586	1735130
	Male	1567736	1253235	1299363	1058382	1685485
Unknown tags^1^	Female	30.93	29.15	25.46	22.18	30.96
	Male	28.91	23.31	25.49	20.85	30.07
Distinct unknown tags	Female	44985	57011	44156	37992	47894
	Male	42744	36131	39384	33696	42548
Distinct unknown tags^1^	Female	53.66	52.71	50.44	48.96	58.31
	Male	53.73	49.99	50.09	48.96	52.78

1 percentage of clean tags (%).

2 percentage of reference unigenes (%).

3 thoraxes and abdomens.

To evaluate the DGE data, we analyzed the distribution of clean tags in each library. Genes with more than 100 copies constituted more than 77.94% of the clean tags but less than 7.04% of the genes (Figs. S3 and S4). In contrast, genes with low expression levels (fewer than five copies) represented many distinct clean tags in each library.

### Differentially Expressed Genes between Tissues

**Figure 8:**
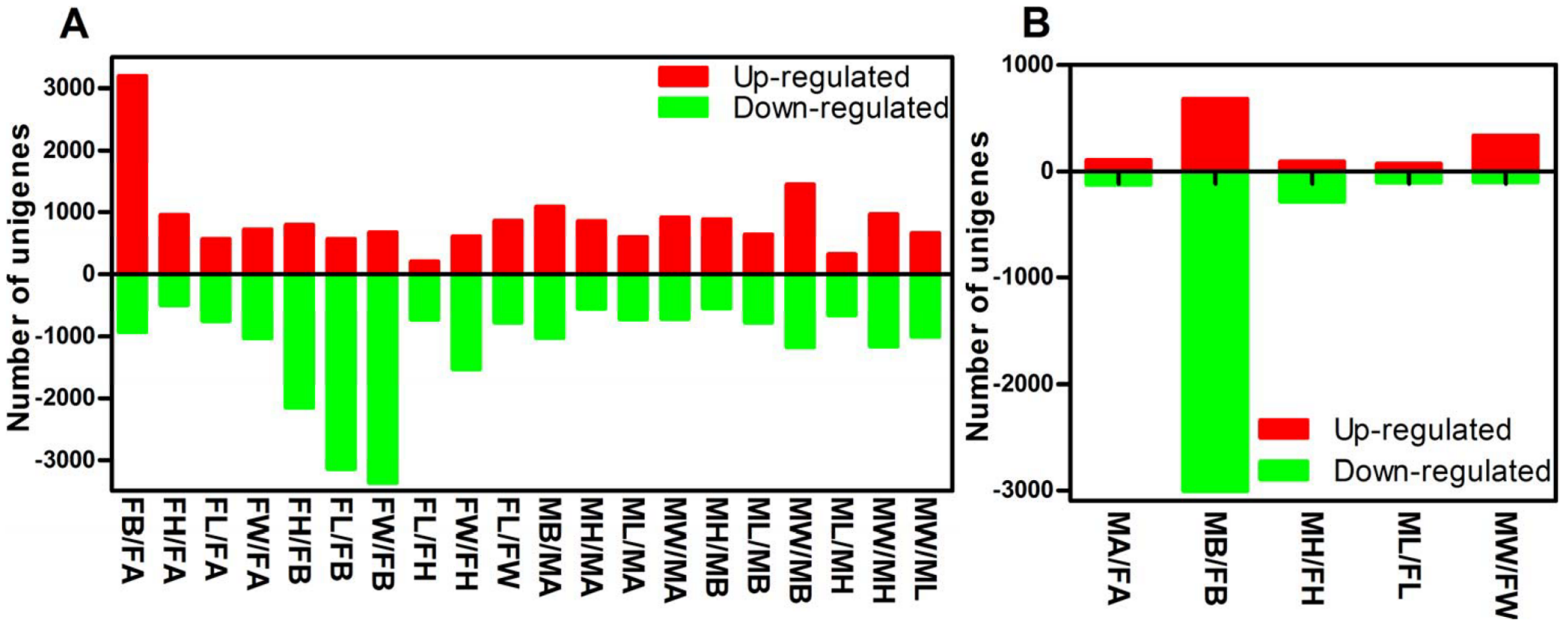
Differentially-expressed unigenes between different tissues of *Chrysopa pallens*. (A) Numbers of up- and down-regulated unigenes in each pair-wise comparison of tissues. (B) Numbers of up- and down-regulated unigenes compared between female and male tissues. FA: female antennae, FH: female heads without antennae, FL: female legs, FW: female wings, FB: female thoraxes and abdomens, MA: male antennae, MH: male heads without antennae, ML: male legs, MW: male wings, MB: male thoraxes and abdomen.

In this study, pair-wise comparison of each tissue within each sex against all the other tissues, respectively, was used to determine the gene expression. Compared with other tissues of each sex, between 439 and 1030 unigenes (females, 439–1030; males, 552–1026) were up-regulated in antennae (Table 3, Fig. 8). Among the 10 most up-regulated unigenes in these eight comparisons (after duplicates were removed), only 11 genes were up-regulated: seven olfactory-related genes (*OBPs* and *CSPs*), a *luciferase*, a *P450*, an *alpha*-*amylase*, and an *extradiol ring-cleavage dioxygenase* (Table S2). According to the GO classification, most of the gene sets that were up-regulated were related to transport. Most of the olfactory and circadian genes were up-regulated in antennae, the center of olfaction.

**Table 3:** Differentially-expressed unigenes between different tissues of *Chrysopa pallens.*

Tissues	FA	FB	FH	FL	FW	MA	MB	MH	ML	MW
**FA**	―	3191	953	568	720	104	N	N	N	N
**FB**	928	―	794	571	673	N	676	N	N	N
**FH**	439	2152	―	208	608	N	N	91	N	N
**FL**	748	3143	726	―	859	N	N	N	68	N
**FW**	1030	3367	1532	775	―	N	N	N	N	331
**MA**	120	N	N	N	N	―	1086	852	597	910
**MB**	N	3004	N	N	N	1026	―	882	637	1442
**MH**	N	N	284	N	N	552	547	―	321	967
**ML**	N	N	N	101	N	718	774	658	―	664
**MW**	N	N	N	N	98	715	1177	1163	1005	―

The number of up-regulated unigenes is listed in the upper right of the table, and the number of down-regulated genes is listed in the bottom left for each tissue comparison.

N: not compared. FA: female antennae, FH: female heads without antennae, FL: female legs, FW: female wings, FB: female thoraxes and abdomens, MA: male antennae, MH: male heads without antennae, ML: male legs, MW: male wings, MB: male thoraxes and abdomen.

When body (thorax and abdomen) tissues were compared with other tissues of each sex, between 547 and 3367 (females, 2152–3367; males, 547–1177) unigenes were up-regulated (Table 3, Fig. 8). Among the top 10 up-regulated unigenes in the comparisons, 11 genes, including a *eupolytin*, a *vitellogenin receptor*, two *enteropeptidases*, two *hemolymph lipopolysaccharide*-*binding proteins*, two *chymotrypsins*, a *lysozyme*, and three *cellular FABPs* were identified (Table S3). Most of the up-regulated genes were involved in the GO metabolic process. Most of the genes related to metabolic process and digestion were more abundant in the body.

In the comparative analysis between the heads and other tissues of each sex, a range from 658 to 1532 (females, 726–1532; males, 658–1163) unigenes were up-regulated (Table 3, Fig. 8). Fifteen genes were identified from the top 10 up-regulated unigenes in the comparisons: a *serine proteinase inhibitor*, three *glucose dehydrogenases*, a *flagelliform silk protein*, a *calmodulin-binding protein*, two *lysozymes*, a *chymotrypsin*, a *venom allergen*, an *ultraviolet-sensitive opsin*, two *cuticular proteins*, a *P450*, and a *transient receptor potential channel* (Table S4). Base on the GO functional classifications, most gene sets were correlated to gene expression and transport.

In legs, as compared with other tissues of each sex, as few as 208 and as many as 1005 (females, 208–775; males, 321–1005) unigenes had significantly higher expressions (Table 3, Fig. 8). Among the top 10 up-regulated unigenes in the comparisons, 25 unigenes were identified: three *glucose dehydrogenases*, a *4-hydroxyphenylpyruvate dioxygenase*, a *serine proteinase inhibitor*, a *trimeric intracellular cation channel*, a *CSP*, two *cuticular proteins*, a *facilitated trehalose transporter*, two *pro-phenol oxidases*, a *long chain fatty acids protein*, a *calcium-independent phospholipase*, an *ionotropic glutamate receptor*, a *P450*, a *vitellogenin*, a *allantoicase*, a *ultraviolet-sensitive opsin*, a 1,*6-bisphosphate aldolase*, a *progestin and adipo Q receptor*, a *chitin deacetylase*, a *cellular FABP-like protein*, and a *glutamate receptor interacting protein* (Table S5). Most of the unigene sets enriched in GO process were involved in metabolic processes. Some of these up-regulated genes were related to the respiratory electron transport chain, which may be involved in providing energy for movement.

Differences in the gene-expression profiles between wings and other tissues of each sex, from 608 to 1442 (females, 608–859; males, 664–1442) unigenes were notably up-regulated (Table 3, Fig. 8). An analysis of the top 10 up-regulated unigenes in the comparisons found that nine unigenes had defined functions, including two *P450s*, one *nose resistant to fluoxetine protein*, two *cuticular proteins*, a *lysozyme*, a *polyprotein*, a *glucose dehydrogenase*, and an *extradiol ring-cleavage dioxygenase* (Table S6). Most of up-regulated unigenes were related to gene expression and metabolic process in females and to transport and metabolic process in males.

Equivalent tissues were also compared between females and males (Table. 3, Fig. 8). Comparing MA and FA, two signal transduction genes, *calmodulin-binding protein* and *G-protein coupled receptor*, were up-regulated in the FA library, and an *OBP gene* was up-regulated in MA. Two *CSPs* gene were up-regulated in FW relative to MW (Table S7 and S8).

### Quantitative Real-time PCR Validation

**Figure 9:**
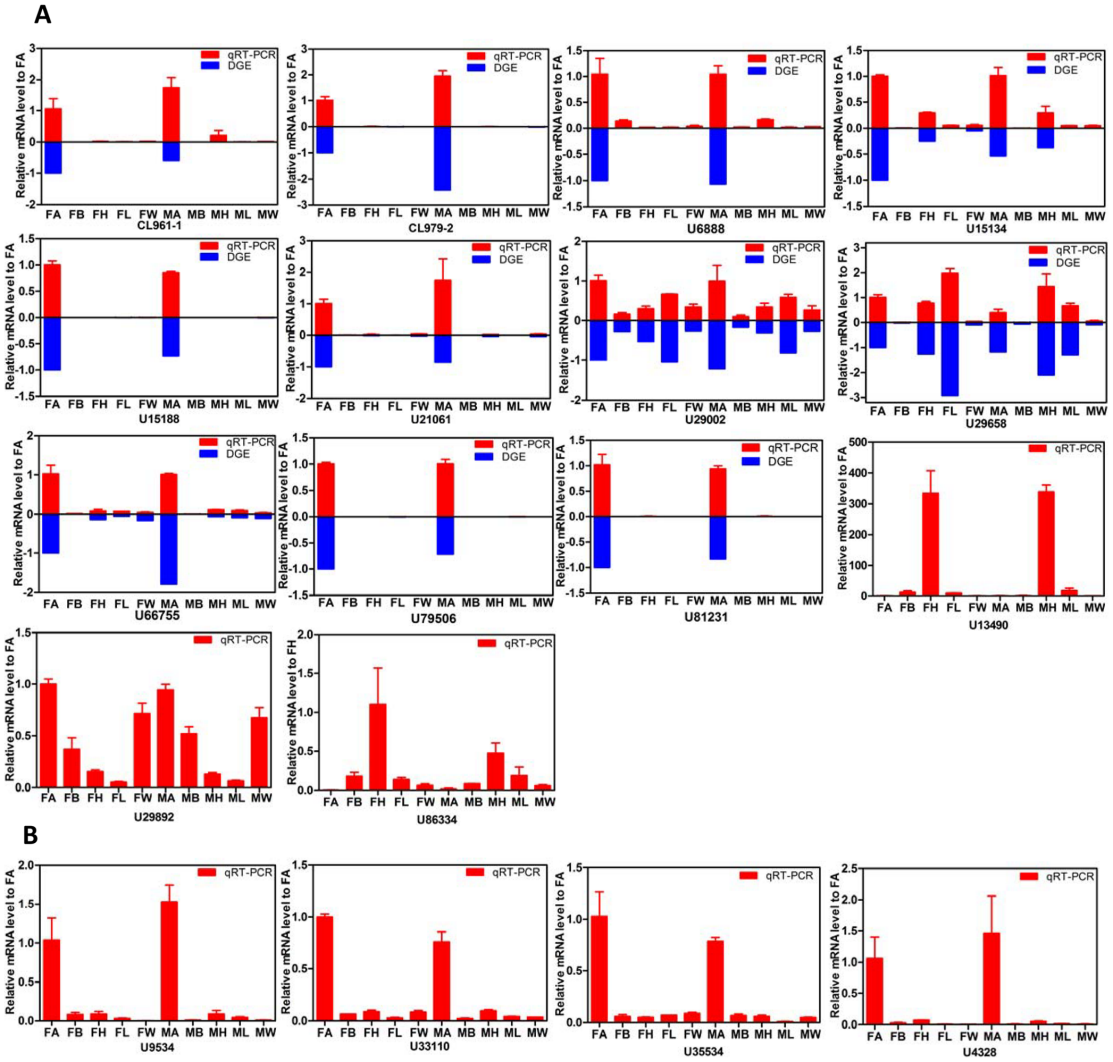
Verification of 14 odorant-binding protein (OBP) genes in tissues of female and male *Chrysopa pallens*. (A) Gene expression analysis of 14 OBP genes for which DGE data were available. Blue, DGE data; red, qRT-PCR results. (B) Gene expression analysis of four OR genes. The relative expression levels were normalized by *GTP-binding protein* (*U15543*) and *ribosomal protein* (*U29931*) Error bars represent standard error.

According to the DGE data, most of the *OBP*s and all of *OR* were abundant mainly in antennae. Among the 14 *OBP* genes, all had more than 10 TPMs (transcripts per million clean tags). We examined the relative expressions of these 14 *OBP* genes. The qRT-PCR results for these genes were consistent with the DGE results (Fig. 9).

By comparing the tissues between females and males, three (*CL961-1*, *U6888*, and *U81231*) had antennal specific expressions and seven (*CL979-2*, *U15134*, *U15188*, *U21061*, *U66755*, and *U79506*) were enriched in antennae. *U29002* and *U29658*showed broad expression profiles with higher expression levels in legs. Interesting, two *OBP*s displayed head enrichment.

## Discussion

*C. pallens* is one of the most important natural enemies of insect pests in China. Until now, no transcriptome information has been available for Neuroptera and sequence data for *C. pallens* were scarce, so more data was a research priority for investigating gene function in this species. In this study, a reference transcriptome was completed, yielding 4.89 Gb of transcriptome data and 56.85 Mb of gene expression tags, using next-generation sequencing technology. To our knowledge, this is the first report of whole transcriptome and DGE profile information for *C. pallens* and could serve as a valuable resource leading ultimately to more effective application of this insect in pest control.

Transcriptome *de novo* assembly was carried out with short reads because of the lack of *C. pallens* genome sequences. In this study, the N_50_ of the unigenes was 1,142 bp, much higher than in other studies [27], [32], suggesting high quality sequencing and assembly. In the transcriptome annotation, only 40.73% of 50,396 unigenes had matches in the NR database, and 12.32% could be annotated to one or more GO terms, indicating that large numbers of the unigenes were either non-coding or specific to *C. pallens*.

Using BLASTX annotation of the *C. pallens* transcriptome, we found that *C. pallens* shared more similarity with *T. castaneum* than with the other species examined, with a significant percentage (35.34%) of highest-similarity sequences. There were 28,690 protein sequences for *Anopheles gambiae* and 27,406 for *T. castaneum*, much more than for the other species. Nevertheless, only 4.37% of the *A. gambiae* were most homologous, much lower than the value for *T. castaneum*. The similarity between *C. pallens* and *T. castaneum* transcriptomes reflected the relatively close phylogenetic relationship between their orders (Neuroptera and Coleoptera).

To analyze the gene expression information, 10 DGE libraries of different tissues were constructed. Most of the differentially-expressed genes were up-regulated in FB. The majority of up-regulated genes were involved in metabolic processes, suggesting that the thorax and abdomen have enhanced metabolic activities, perhaps because these tagmata are the most important sites of digestion, circulation, and reproduction. Compared with other tissues, the *period*, *timeless*, and *clock* genes, which are involved in circadian rhythms, were more up-regulated in heads, antennae and, particularly, in wings, which are the most important organs for circadian rhythms in *C. pallens* [38]–[40].

The insect olfactory system is a highly specific and sensitive chemical detector essential for feeding, mating, and finding oviposition sites. To better understand the sophisticated olfactory system of *C. pallens*, 66 putative chemoreception genes(including *OBPs*, *CSPs*, *ORs*, *IRs*, and *SNMPs*) were identified from our transcriptome. This number is more than the numbers found in *Cnaphalocrosis medinalis* (22) [41] and *Cotesia vestalis* (28) [42], but less than that of *Spodoptera littoralis* (82) [30], *Manduca sexta* (94) [43] and *Helicoverpa armigera* (99) [34].

OBPs are thought to aid in capture and transport of odorants and pheromones to chemoreceptors [44], [45], which were discovered in the early 1980s in the giant moth *Antheraea polyphemus* [10]. On the basis of BLASTX results, all 14 candidate *OBPs* were classified into two groups: 12 *OBPs* and 2 *PBPs*. Base on the *OBP* sequences, where only the cysteine motif defines the relatedness, insect OBPs have been further grouped into: Classic OBPs (with 6 conserved cysteines), Plus-C OBPs (with more than six conserved cysteines) and Minus-C (with only four conserved cysteines) [1], [46]–[48]. According to DGE and qRT-PCR results (Fig 9 and Table S1), *OBPs* and *ORs* were abundant mainly in antennae, consistent with studies on *OBPs* and *ORs* in other insects 46,49. Three of 14 *OBPs*, which had antennae-specific expressions, may play important roles in pheromone detection. Interestingly, two putative *OBP*s (*U29002* and *U29658*) were expressed at very high levels in legs, and two *OBP*s showed head enrichment. This high expression might suggest the unique functions in chemoreception.

As other soluble secreted proteins, CSPs are also found in the sensillum lymph, but their roles in olfaction remain elusive. Insect CSPs were also known as OS-D-like proteins [48] or sensory appendage proteins (SAPs) [35] before being named CSPs [47]. In our study, most of the *CSPs* showed broad expression profiles which were consistent with previous studies on these genes in other insects [50]–[52]. In brief, antennae-enriched OBPs and CSPs may be involved in *C. pallens* identifying and binding volatile from pests, compatriots or pest-damaged plants.

Antennae-restricted expression is a useful criterion to identify genes involved in specific olfactory functions, including ODEs. Because the sequences of these transcripts were so short in the transcriptome, most have not been detected by DGE. Interestingly, 12 of the 60 *P450s* were clearly enriched in or restricted to antennae, suggesting an ODE function. In addition, *CL147-6* and *U30217* were related to the CYP4A subfamily of P450s (Fig. 7). Several genes in this family are olfaction specific and also expressed in olfactory sensilla [53].

To better understand the OBPs and CSPs, phylogenetic trees of each of these genes were constructed. Three antennae enriched candidate OBPs showed higher homology to *A. pisum* OBPs (Fig. 5 and Fig. 9). Because aphids are the major prey of *C. pallens*, the higher homology of candidate OBPs between *C. pallens* and *A. pisum* OBPs may play an important role in hunting. In the CSPs phylogenetic tree, most of the CSPs from the same species formed a clade. Nevertheless, eight CSPs from five of the six species clustered together (Fig. 6). This high conservation of CSPs may indicate the multiple copies formed after these insect orders diverged from one another and that these proteins may have crucial functions. Competitive fluorescence binding assays, fluorescent *in situ* hybridization, RNAi, and behavioral bioassays may be used to characterize these olfaction-related genes in further studies [54], [55].

The DGE results were consistent with the major functions of different tissues, most of the olfaction genes were up-regulated in male and female antennae, the center of olfaction. The antennae-enriched P450s may play roles in digesting poisonous odors to protect the sensitivity of sensory neurons [53], [56]. Additionally, three insecticide-resistance-related genes, including two *P450s*, were enriched in wings, indicating that wings may play an important role in insecticide resistance. Some genes related to energy generation and transmission, three *glucose dehydrogenases*, an *ionotropic glutamate receptor*, and a *facilitated trehalose transporter*, were up-regulated in legs, consistent with their important function in movement.

### Conclusions

Using next-generation sequencing technology, we provided large-scale sequence information for *C. pallens* and identified 14 *OBPs*, 22* CSPs*, 60 *P450s*, 16 *IRs*, 14 *ORs*, 2 *SNMPs*, 54 *CXEs,* and 21 *AOXs*. This large number of insect chemosensory genes will provide the basis for functional studies. Together with the evolutionary analysis, the results provide new insights into the differentiation and evolution of insect OBPs and CSPs, as well as data for further studies on non-olfaction genes in *C. pallens* and other Neuropterans. Understanding how structure, function, and gene expression interact in the olfactory systems of predatory insects can inform the development of new semiochemical tools that will improve biological control in sustainable agriculture.

## Methods

### Insects

The *C. pallens* used in this experiment were provided by the Plant Protect & Environment Protect Research Institute, Beijing Academy of Agriculture and Forestry Sciences, China. Experimental insects were the offspring of a single female and reared in the laboratory on *Acyrthosiphon pisum*. Rearing conditions were 25±1°C, a 14∶10 h light/dark (L:D) photoperiod, and 65±5% relative humidity (RH). Pupae were kept in separate cages for eclosion, after which adults were supplied with *A. pisum*. Thirty 3-day-old virgin females and males, respectively, were used to collect female antennae (FA), heads (FH), wings (FW), legs (FL) and thoraxes and abdomens (FB) and male antennae (MA), heads (MH), wings (MW), legs (ML) and thoraxes and abdomens (MB). Tissue samples were kept at −80°C until RNA isolation.

### cDNA Library Preparation and Illumina Sequencing for Transcriptomes

Total RNA was extracted by SV Total Isolation System (Promega, Madison, WI, USA) following the manufacturer’s instructions. cDNA library construction and Illumina sequencing of the samples were performed at Beijing Genomics Institute – Shenzhen, Shenzhen, China [57]. Briefly, oligo(dT) beads were used to isolate poly (A) mRNA from 20 µg of pooled total RNA(FA : FH : FW : FL : FB : MA : MH : MW : ML : MB at 5∶1:1∶1:1∶5:1∶1:1∶1 ratio). To interrupt mRNA into short fragments, fragmentation buffer and divalent cations were used at 94°C for 5 min. Using these short fragments as templates, random hexamer-primers were used to synthesize first-strand cDNA. Second-strand cDNA was generated using buffer, dNTPs, RNAseH, and DNA polymerase I. After end-repair and ligation of adaptors, the products were amplified by PCR and purified with QIAquick PCR extraction kit (Qiagen, Venlo, Netherlands) and resolved with EB buffer for end reparation and adding poly (A). Then, the short fragments connected to sequencing adapters and detected by agarose gel electrophoresis were selected as templates for PCR amplification and sequencing using Illumina HiSeq™ 2000 (San Diego, CA, USA).

### Assembly and Function Annotation

Transcriptome *de novo* assembly was carried out with the short-read assembly program Trinity [58], which generated two classes of unigenes: clusters (prefix CL) and singletons (prefix unigene). Finally, unigenes larger than 150 bp were first aligned by BLASTX to protein databases including Nr, Swiss-Prot, KEGG and COG (e-value<10^−5^) and by BLASTN to the NCBI nucleotide databases (Nt; e-value <10^−5^) to retrieve proteins with the highest sequence similarity with the given unigenes along with their protein functional annotations.

To validate the assembly results, 14 OBPs and 22 CSPs were confirmed by end-to-end RT-PCR using specific primers designed using Primer Premier 5.0 (Table S9). The PCR products were purified using the Wizard® SV Gel and PCR Clean-Up System (Promega) and then subcloned into a T/A plasmid using the pEASY-T3 cloning vector system (TransGene, Beijing, China) following manufacturer’s instructions. In this study, the clean read and computationally assembled sequences were submitted and available from the NCBI/SRA data base and NCBI/TSA repository (SRA experiment accession number: SRX219870, TSA accession number: GAGF01000001 - GAGF01050315, BioProject accession number: PRJNA186574).

### Sequence Alignment and Phylogenetic Analysis

The amino acid sequence alignment of the candidate OBPs and CSPs were performed using CLUSTALX 2.0 [59] and then arranged by Jalview 2.4.0 b2 [60]. The 14 OBP, 22 CSP, and 47 P450 conceptually-translated sequences from the *C. pallens* transcriptome, along with OBPs, CSPs, and P450s from other insect species, were used to construct three phylogenetic trees based on the amino sequences. The OBP data set contained OBPs from five other insect species (46 from *T. castaneum*, 46 from *Drosophila melanogaster*, 43 from *Bombyx mori*, 13 from *Acyrthosiphon pisum*, and 27 from *Apis mellifera*) (Table S10). The CSP data set contained CSPs from five other insect species (20 from *T. castaneum*, 19 from *D. melanogaster*, 16 from *B. mori*, 6 from *A. pisum*, and 6 from *A. mellifera*) (Table S11). The signal peptide predicted by SignalIP 4.0 (http://www.cbs.dtu.dk/services/SignalP/) was deleted from the amino acid sequences of OBPs and CSPs before phylogenetic analyses. The P450 data set contained 82 P450 sequences from *B. mori* [14]. Amino acid sequences for each protein were aligned using ClustalX 1.83 [61]. Neighbor-joining trees were produced using MEGA5 [62] with Poisson correction of distances, and 1000 neighbor-joining bootstrap replicates were performed.

### DGE Library Preparation and Sequencing

Six µg total RNA were extracted from FA, FH, FL, FW, FB, MA, MH, ML, MW, and MB as described above, and DGE library preparation and sequencing followed previously-described protocols [32]. mRNA were purified by using Oligo (dT) magnetic beads adsorption, 5′ and 3′ ends of tags were generated by endonuclease *MmeI*, *NlaIII* and *DpnII* (Table S12). Illumina adaptors(sense: 5′ACACTCTTTCCCTACACGACGCTCTTCCGATC3′ and 5′GATCGGAAGAGCGGTTCAGCAGGAATGCCGAG3′) were ligated to the sticky 5′ and 3′ ends of tags, respectively. At last, the short fragments connected to sequencing adapters were selected as templates for PCR amplification and sequencing using Illumina HiSeq™ 2000.

### Analysis and Gene Expression Annotation of DGE Tags

DGE tag mapping followed previously-described protocols [32]. The raw sequences were filtered to clean tags, and were mapped to the transcriptome, used as reference sequences containing all the possible clean tags containing CATG and 17 bases length sequences of the reference gene sequences. The number of unambiguous clean tags for each gene was calculated and then normalized to TPM (number of transcripts per million clean tags) [63], [64].

### Screening of Differentially Expressed Genes

A rigorous algorithm was developed to identify differentially expressed genes (DEGs) between the different tissues of *C. pallens*, referring to the method described previously [65]. *P* Value corresponds to differential gene expression test. False discovery rate (FDR) was used to determine the threshold of *P* value in multiple tests and analysis through manipulating the FDR value. We use FDR ≤0.001 and the absolute value of log_2_ ratio ≥1 as the threshold to judge the significance of gene expression difference [66].

### GO Functional Enrichment Analysis for DGEs

In gene expression profiling analysis, GO enrichment analysis of functional significance followed previously-described protocols [32]: applies hypergeometric test to map all differentially expressed genes to terms in GO database, looking for significantly enriched GO terms in DEGs comparing to the genome background. The calculating formula is:
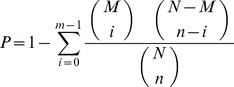



In this equation, *N* is the number of all genes with GO annotation; *n* is the number of DEGs in *N*; *M* is the number of all genes that are annotated to the certain GO terms; *m* is the number of DEGs in *M*. For GO enrichment analysis, all of the *P* values were calculated with Bonferroni correction. We selected a corrected *P* value ≤0.05 as a threshold to determine significant enrichment of the gene sets.

### Quantitative Real-time PCR Validation

Total RNA was extracted as described above. The concentration of each RNA sample was standardized to 0.5 µg/µL for reverse transcription. cDNAs were synthesized using Reverse Transcription System (Promega) according to the manufacturer’s protocol. The results were normalized by the internal controls *GTP-binding protein* (*U15543*) and *ribosomal protein* (*U29931*). qRT-PCR primers were designed based on the nucleotide sequences of the chosen unigenes using Beacon Designer 7.7. Primers and unigenes used in qRT-PCR are listed Table S13. qRT-PCR reactions were run in triplicate (technical repeats) with three independent biological replicates. The quantitative validation was analyzed by a relative quantitative method (2^–△△CT^) [67].

## Supporting Information

Figure S1 COG classification of *Chrysopa pallens* unigenes. This figure shows the COG classifications of the unigene BLASTX results against the COG database. The X-axis shows the function class of unigenes. The Y-axis shows the number of unigenes.
(TIF)

Figure S2 Different components of the raw tags in each *Chrysopa pallens* tissue sample. The percentages of tags containing Ns, only adaptors, a tag copy number <2, and clean tags are shown. FA: female antennae, FH: female heads without antennae, FL: female legs, FW: female wings, FB: female thoraxes and abdomens, MA: male antennae, MH: male heads without antennae, ML: male legs, MW: male wings, MB: male thoraxes and abdomen.
(TIF)

Figure S3 Distribution of total and distinct clean tags in each female *Chrysopa pallens* sample. Numbers in square brackets indicate the copy number ranges for each tag category. Data in parentheses indicate the numbers and percentages of each category of tags. (A) Distribution of total clean tags. (B) Distribution of distinct clean tags.
(TIF)

Figure S4 Distribution of total and distinct clean tags in each male *Chrysopa pallens* sample. Numbers in square brackets indicate copy number ranges for each tag category. Data in parentheses indicate the numbers and percentages of each category of tags. (A) Distribution of total clean tags. (B) Distribution of distinct clean tags. FA: female antennae, FH: female heads without antennae, FL: female legs, FW: female wings, FB: female thoraxes and abdomens, MA: male antennae, MH: male heads without antennae, ML: male legs, MW: male wings, MB: male thoraxes and abdomen.
(TIF)

Table S1 BLASTX results and digital gene expression profiles of candidate *OBPs*, *CSPs*, *P450s*, *IRs*, *ORs*, *CXEs* and *AOXs* from *Chrysopa pallens.* Information includes gene ID in this transcriptome, open reading frame length, gene name, accession number, species, E-value, identity to other proteins, and number of transcripts per million clean tags (TPM).
(XLSX)

Table S2 Top 10 most up-regulated unigenes in antennae compared with other tissues, including gene name, expression ratio, GO process, and annotation result.
(XLSX)

Table S3 Top 10 most up-regulated unigenes in the thorax and abdomen compared with other tissues, including gene name, expression ratio, GO process, and annotation result.
(XLSX)

Table S4 Top 10 most up-regulated unigenes in heads compared with other tissues, including gene name, expression ratio, GO process, and annotation result.
(XLSX)

Table S5 Top 10 most up-regulated unigenes in legs compared with other tissues, including gene name, expression ratio, GO process, and annotation result.
(XLSX)

Table S6 Top 10 most up-regulated unigenes in wings compared with other tissues, including gene name, expression ratio, GO process, and annotation result.
(XLSX)

Table S7 Top 10 most up-regulated unigenes in male compared with female tissues, including gene name, expression ratio, GO process and annotation result.
(XLSX)

Table S8 Top 10 most up-regulated unigenes in female compared with male tissues, including gene name, expression ratio, GO process and annotation result.
(XLSX)

Table S9 Primers and unigenes used in end-to-end RT-PCR validation, including unigene names and primer sequences.
(XLSX)

Table S10 Odorant-binding proteins used in phylogenetic tree construction, including protein name and GenBank accession number.
(DOCX)

Table S11 Chemosensory proteins used in phylogenetic tree construction, including protein name and GenBank accession number.
(DOCX)

Table S12 Recognition sites of several endonucleases on cDNA in the sample prepared for the construction of DGE libraries.
(DOCX)

Table S13 Primers and unigenes used in quantitative real-time PCR validation, including unigene names and primer sequences.
(XLSX)
