# Supplementary figures and images for: First Transcriptome and Digital Gene Expression Analysis in Neuroptera with an Emphasis on Chemoreception Genes in Chrysopa pallens (Rambur)
Source: PLoS One. 2013 Jun 27;8(6):e67151. doi: 10.1371/journal.pone.0067151 (PMC3694914; doi:10.1371/journal.pone.0067151)

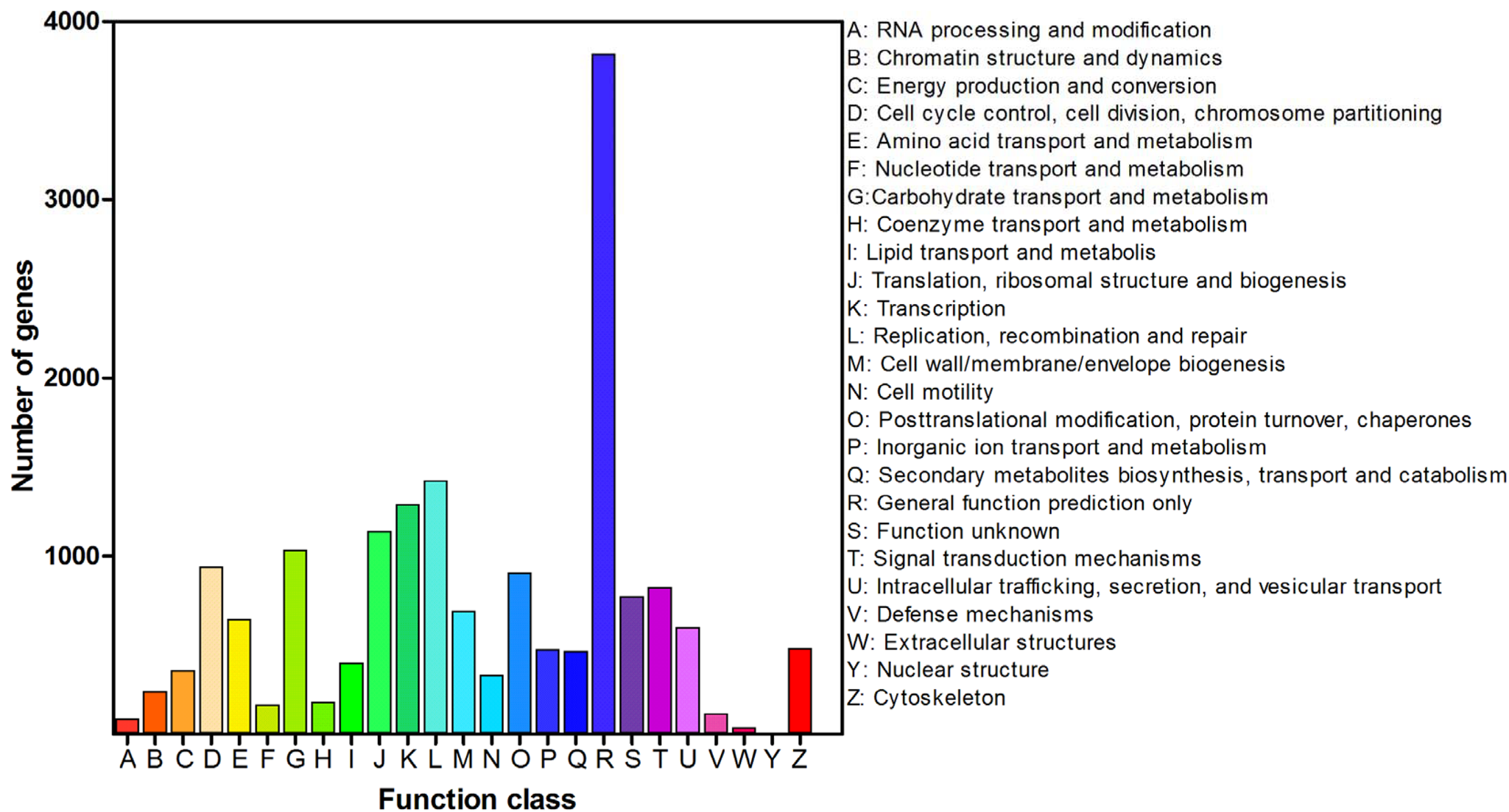

Supplement: Figure S1 — COG classification of Chrysopa pallens unigenes. This figure shows the COG classifications of the unigene BLASTX results against the COG database. The X-axis shows the function class of unigenes. The Y-axis shows the number of unigenes. (TIF) [file pone.0067151.s001.tif]

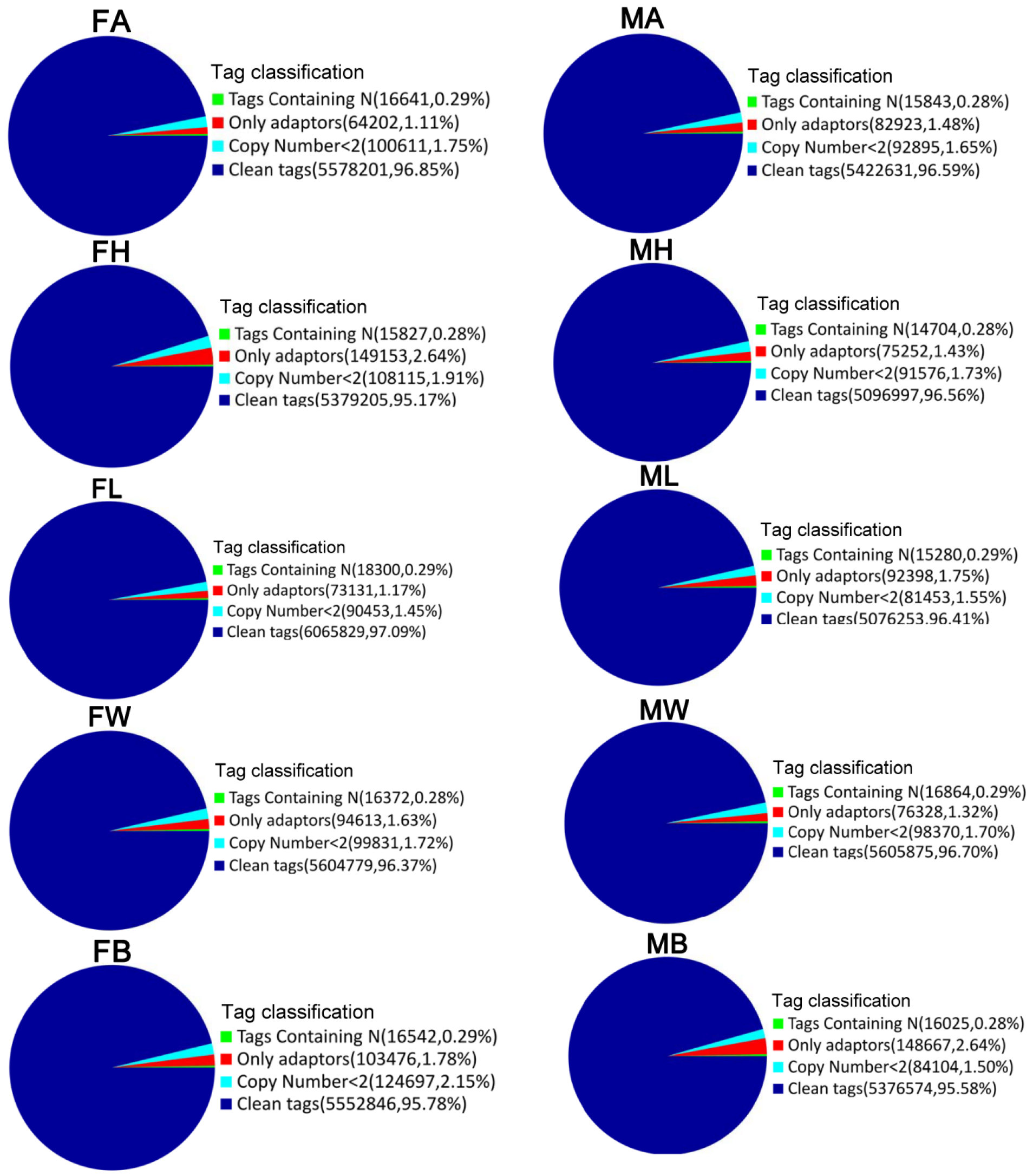

Supplement: Figure S2 — Different components of the raw tags in each Chrysopa pallens tissue sample. The percentages of tags containing Ns, only adaptors, a tag copy number <2, and clean tags are shown. FA: female antennae, FH: female heads without antennae, FL: female legs, FW: female wings, FB: female thoraxes and abdomens, MA: male antennae, MH: male heads without antennae, ML: male legs, MW: male wings, MB: male thoraxes and abdomen. (TIF) [file pone.0067151.s002.tif]

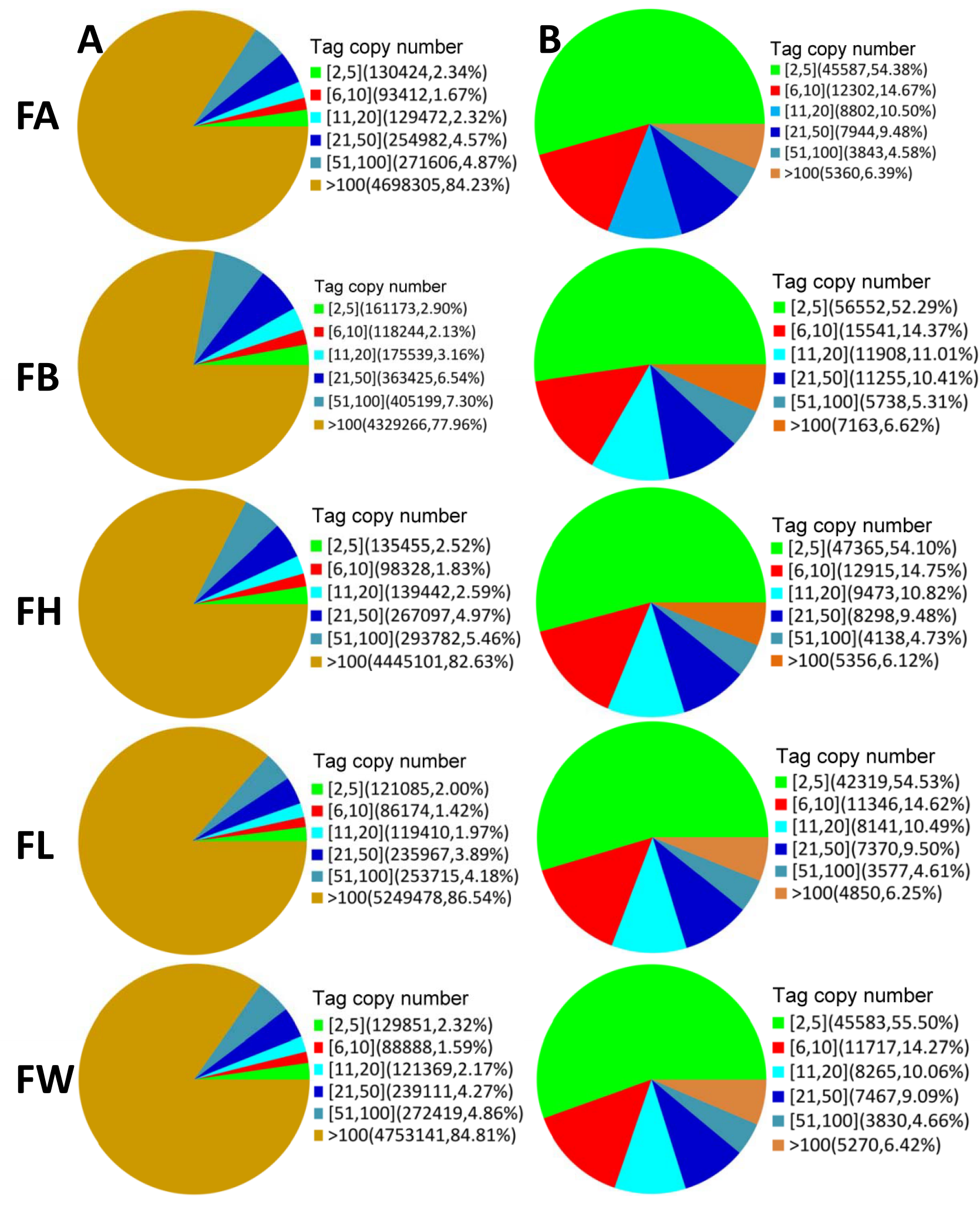

Supplement: Figure S3 — Distribution of total and distinct clean tags in each female Chrysopa pallens sample. Numbers in square brackets indicate the copy number ranges for each tag category. Data in parentheses indicate the numbers and percentages of each category of tags. (A) Distribution of total clean tags. (B) Distribution of distinct clean tags. (TIF) [file pone.0067151.s003.tif]

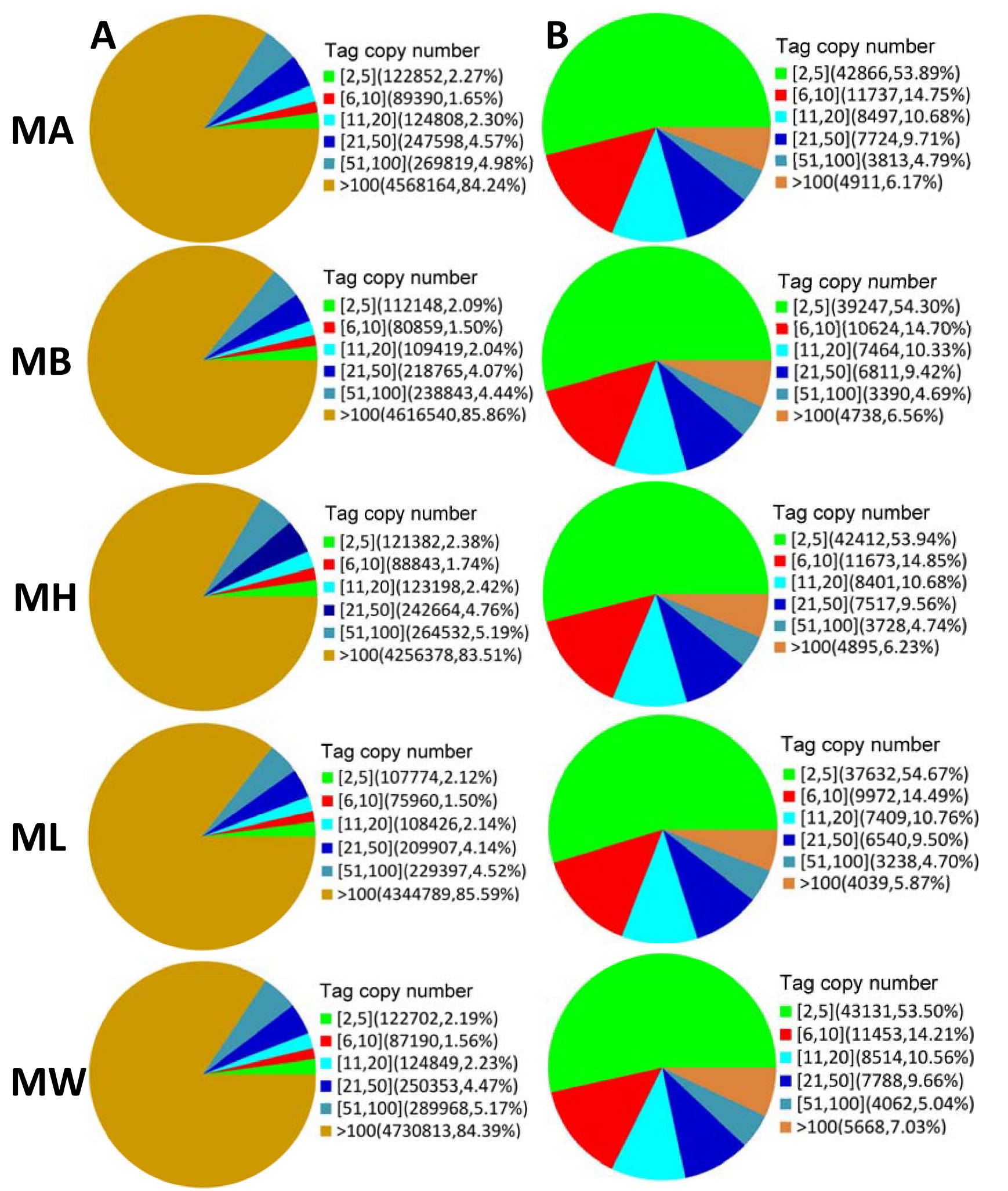

Supplement: Figure S4 — Distribution of total and distinct clean tags in each male Chrysopa pallens sample. Numbers in square brackets indicate copy number ranges for each tag category. Data in parentheses indicate the numbers and percentages of each category of tags. (A) Distribution of total clean tags. (B) Distribution of distinct clean tags. FA: female antennae, FH: female heads without antennae, FL: female legs, FW: female wings, FB: female thoraxes and abdomens, MA: male antennae, MH: male heads without antennae, ML: male legs, MW: male wings, MB: male thoraxes and abdomen. (TIF) [file pone.0067151.s004.tif]
