# Supplementary material for: First Transcriptome and Digital Gene Expression Analysis in Neuroptera with an Emphasis on Chemoreception Genes in Chrysopa pallens (Rambur)
Source: PLoS One. 2013 Jun 27;8(6):e67151. doi: 10.1371/journal.pone.0067151 (PMC3694914; doi:10.1371/journal.pone.0067151)
Supplement: Table S10 — Odorant-binding proteins used in phylogenetic tree construction, including protein name and GenBank accession number. (DOCX) [file pone.0067151.s014.docx]

**Table S10.** OBPs used in phylogenetic tree construction.

| OBP | Acc No. | OBP | Acc No. |
| --- | --- | --- | --- |
| ApisOBP01 | CAR85628 | AmelOBP02 | NP_001011591 |
| ApisOBP02 | CAR85629 | AmelOBP03 | ABD92639 |
| ApisOBP03 | CAR85630 | AmelOBP04 | NP_001011589 |
| ApisOBP04 | CAR85631 | AmelOBP05 | NP_001011588 |
| ApisOBP05 | CAR85632 | AmelOBP06 | NP_001011593 |
| ApisOBP06 | CAR85633 | AmelOBP07 | ABD92640 |
| ApisOBP07 | CAR85634 | AmelOBP08 | NP_001164515 |
| ApisOBP08 | CAR85635 | AmelOBP09 | ABD92641 |
| ApisOBP09 | CAR85636 | AmelOBP10 | ABD92642 |
| ApisOBP10 | CAR85637 | AmelOBP11 | ABD92643 |
| ApisOBP11 | CAX63068 | AmelOBP12 | ABD92644 |
| ApisOBP12 | CAX63069 | AmelOBP13 | ABD92645 |
| ApisOBP13 | CAX63070 | AmelOBP14 | ABD92646 |
| AmelASP01 | AF393494 | AmelOBP15 | ABD92647 |
| AmelASP02 | AF393493 | AmelOBP16 | ABD92648 |
| AmelASP03 | AF481963 | AmelOBP17 | ABD92649 |
| AmelASP04 | AF393495 | AmelOBP18 | ABD92650 |
| AmelASP05 | AF393497 | AmelOBP19 | ABD92651 |
| AmelASP06 | AF393496 | AmelOBP20 | ABD92652 |
| AmelOBP01 | NP_001011590 | AmelOBP21 | ABD92653 |

All TcasOBP sequences are from Foret and Maleszka, 2006, and all DmelOBPs sequences are from J.-J. Zhou, 2010.
