# Supplementary material for: First Transcriptome and Digital Gene Expression Analysis in Neuroptera with an Emphasis on Chemoreception Genes in Chrysopa pallens (Rambur)
Source: PLoS One. 2013 Jun 27;8(6):e67151. doi: 10.1371/journal.pone.0067151 (PMC3694914; doi:10.1371/journal.pone.0067151)
Supplement: Table S11 — Chemosensory proteins used in phylogenetic tree construction, including protein name and GenBank accession number. (DOCX) [file pone.0067151.s015.docx]

| CSP | Acc No. | CSP | Acc No. | CSP | Acc No. | CSP | Acc No. |
| --- | --- | --- | --- | --- | --- | --- | --- |
| TcasCSP01 | EFA07423 | TcasCSP18 | EFA07570 | DmelCSPA75a | AAF49268 | BmorCSP13 | ABH88206 |
| TcasCSP02 | EFA07420 | TcasCSP19 | EFA07577 | DmelCSPA98a | AAF56814 | BmorCSP14 | ABH88207 |
| TcasCSP03 | EFA07417 | TcasCSP20 | EFA01297 | DmelCSPA7a | AAF46258 | BmorCSP15 | ABH88208 |
| TcasCSP04 | EFA07418 | DmelCSPB42a | AAM70837 | DmelCSPA29a | AAO41170 | BmorCSP16 | ABH88209 |
| TcasCSP05 | EFA07419 | DmelCSPB42b | AAS64791 | DmelCSPA87a | AAF54934 | AmelCSP01 | ABH88169 |
| TcasCSP06 | EFA07421 | DmelCSPB42c | AAS64792 | BmorCSP01 | ABH88194 | AmelCSP02 | ABH88170 |
| TcasCSP07 | EFA07424 | DmelCSPB38a | AAS64728 | BmorCSP02 | ABH88195 | AmelCSP03 | ABH88171 |
| TcasCSP08 | EFA07640 | DmelCSPB38b | AAS64727 | BmorCSP03 | ABH88196 | AmelCSP04 | ABH88172 |
| TcasCSP09 | EFA07422 | DmelCSPB74a | AAZ66059 | BmorCSP04 | ABH88197 | AmelCSP05 | ABH88173 |
| TcasCSP10 | EFA07552 | DmelCSPB93a | AAF55894 | BmorCSP05 | ABH88198 | AmelCSP06 | ABH88174 |
| TcasCSP11 | EFA07563 | DmelCSPB93b | AAN13876 | BmorCSP06 | ABH88199 | ApisCSP01 | NP_001156200 |
| TcasCSP12 | EFA07566 | DmelCSPB53a | AAX52701 | BmorCSP07 | ABH88200 | ApisCSP02 | NP_001119649 |
| TcasCSP13 | EFA07565 | DmelCSPB53b | AAX52702 | BmorCSP08 | ABH88201 | ApisCSP03 | NP_001119650 |
| TcasCSP14 | EFA07564 | DmelCSPA86a | AAZ52549 | BmorCSP09 | ABH88202 | ApisCSP04 | NP_001119651 |
| TcasCSP15 | ABH88188 | DmelCSPA46a | AAZ52819 | BmorCSP10 | ABH88203 | ApisCSP05 | NP_001119652 |
| TcasCSP16 | EFA07567 | DmelCSPA56a | AAZ52804 | BmorCSP11 | ABH88204 | ApisCSP06 | NP_001128404 |
| TcasCSP17 | EEZ99322 | DmelCSPA84a | AAZ52518 | BmorCSP12 | ABH88205 |  |  |

**Table S11.** CSPs used in phylogenetic analysis.
