# Supplementary material for: First Transcriptome and Digital Gene Expression Analysis in Neuroptera with an Emphasis on Chemoreception Genes in Chrysopa pallens (Rambur)
Source: PLoS One. 2013 Jun 27;8(6):e67151. doi: 10.1371/journal.pone.0067151 (PMC3694914; doi:10.1371/journal.pone.0067151)
Supplement: Table S12 — Recognition sites of several endonucleases on cDNA in the sample prepared for the construction of DGE libraries. (DOCX) [file pone.0067151.s016.docx]

**Table S12.** Recognition sites of several Endonuclease on cDNA in sample preparation

| Endonuclease | *Nla*III | *Dpn*II | *Mme*I |
| --- | --- | --- | --- |
| Recognition sites | 5’ …CATG…3’  3’ …GTAC…5’ | 5’ …GATC…3’  3’ …CTAG…5’ | 5’ …TCCRAC(N)_20_ …3’  3’ …AGGYTG(N)_18_ …5’ |
